# Electrospun Nanofibers for the Delivery of Endolysin/Dendronized Ag-NPs Complex Against Pseudomonas aeruginosa

**DOI:** 10.2147/NSA.S498942

**Published:** 2025-02-18

**Authors:** Magdalena Lasak, Małgorzata Łysek-Gładysińska, Karolina Lach, Viraj P Nirwan, Dorota Kuc-Ciepluch, Javier Sanchez-Nieves, Francisco Javier de la Mata, Amir Fahmi, Karol Ciepluch

**Affiliations:** 1 Division of Medical Biology, Jan Kochanowski University in Kielce, Kielce, Poland; 2 Faculty of Technology and Bionics, Rhine-Waal University of Applied Science, Kleve, Germany; 3 Department of Basic Medical Sciences, Faculty of Medical Sciences and Health Sciences, Casimir Pulaski University of Radom, Radom, Poland; 4 Department of Organic and Inorganic Chemistry, Research Institute in Chemistry “Andrés M. del Río” (IQAR), University of Alcalá (UAH), Alcalá de Henares, Spain; 5 Networking Research Center for Bioengineering, Biomaterials and Nanomedicine (CIBER-BBN), Madrid, Spain; 6 Ramón y Cajal Institute of Health Research, (IRYCIS), Madrid, Spain

**Keywords:** nanofibers, dendritic nanoparticles, endolysin, antibacterial agents

## Abstract

**Purpose:**

As bacterial resistance to antibiotics increases, there is an urgent need to identify alternative antibacterial agents and improve antibacterial materials. One is the controlled transport of antibacterial agents that prevents infection with drug-resistant bacteria, especially in the treatment of difficult-to-heal wounds.

**Methods:**

This work presents the use of electrospun PLCL/PVP (poly(L-lactide-co-ε-caprolactone/polyvinylpyrrolidone) nanofibers modified with two agents with antibacterial properties but with different mechanisms of action, that is, dendritic silver nanoparticles (Dend-AgNPs) and endolysin.

**Results:**

The nanomat prepared in this manner showed significant antibacterial activity against antibiotic-resistant Pseudomonas aeruginosa strains, inhibiting their growth and production of key pigments and virulence factors. Moreover, the use of nanofibers as carriers of the selected factors significantly reduced their cytotoxicity towards human fibroblasts.

**Conclusion:**

The results confirmed the possibility of using the presented product as an innovative dressing material, opening new perspectives for the treatment of wounds and combating bacterial infections with drug-resistant bacteria.

## Graphical Abstract

**Figure:**
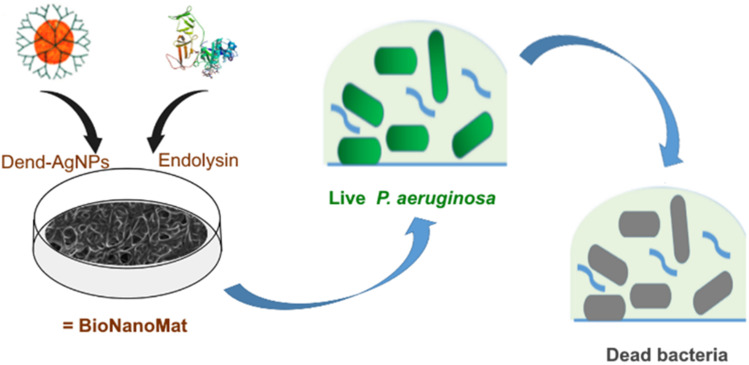

## Introduction

Effective platforms for antimicrobial agents based on nanomaterials should be characterized by special physicochemical properties that allow easy functionalization and immobilization for the transport of compounds with a different spectrum of activities. This type of platform allows better therapeutic effectiveness and biodistribution of drug.^1–4^ Among the most popular nanomaterials, both natural and synthetic nanomaterials exhibit unique physicochemical and biological properties.^4–8^ Synthetic nanomaterials have properties similar to those of natural polymers, but are more popular owing to their satisfactory strength and flexibility, as well as low cytotoxicity and biodegradability. One method for obtaining synthetic nanomaterials with the previously presented properties is electrospinning.^4^,^8–11^ Nanomats based on electrospun nanofibers with a biodegradable and biocompatible poly(L-lactide-co-ε-caprolactone) copolymer have been widely used in the creation of engineering materials in bionanotechnology and medicine, for example, in regenerative medicine, tissue formation, and antimicrobial properties.^5^,^6^,^12^,^13^ Appropriate functionalization with therapeutic agents, such as antibacterial agents, anti-inflammatory agents, anesthetics, growth factors, and antioxidants, alleviates discomfort, while ensuring appropriate conditions for wound healing. However, in addition to the aforementioned features, additional aspects must be considered during the wound healing process. First, the material must be nontoxic, nonimmunogenic, biocompatible, and appropriately conform to the location of its application.^14^,^15^ These aspects are important because open wounds create favorable conditions for the development of pathogens that can infect wounds, impede the healing process, and attack nearby healthy tissues. Thus, incorporation of antimicrobial agents into electrospun nanofibers is an attractive strategy for designing ideal materials to reduce pathogen colonization and promote wound healing. Loading just one type of antimicrobial into a nanofibers does not always provide the expected antimicrobial activity, especially when it comes to fighting drug-resistant bacteria such as gram-negative bacteria. Therefore, combinations of agents in the form of antibacterial complexes are often used to enhance antibacterial effect.^16–19^ This is especially important in third-world countries, where the risk of infection with resistant bacteria is high and results from poorly developed healthcare systems. In many cases, wound formation or surgery often leads to bacterial infections. Undoubtedly, one of the main pressures for the production of nanomaterials with unique antibacterial properties is increasing bacterial resistance to antibiotics.^20–22^ This can be achieved by selecting antibacterial agents with different properties, which together provide a synergistic effect and allow the defeat of drug-resistant bacteria, which, according to the WHO, are responsible for almost five million deaths each year. Therefore, the selection of appropriate ingredients for a antimicrobial nanomaterial is crucial. Among the large libraries of potential agents, metal nanoparticles and antibacterial proteins are worthy of attention.

Dendritic metal NPS, such as silver nanoparticles (AgNPs) decorated with dendrimers or dendrons, in addition to the well-known properties of silver, also support positive charges on the surface of NPs owing to the presence of cationic dendritic systems. This combination facilitates their interaction with the negatively charged outer membrane of bacteria and the many proteins located on their surface.^23–25^ The mechanism of action of AgNPs usually involves the production of reactive oxygen species (ROS) that affect the outer bacterial membrane (increase permeability, cause lipid peroxidation), damage the bacterial cell wall, and can bind or inhibit enzymes. Additionally, reactions with the thiol groups of cysteine and phosphorus compounds influence the processes of bacterial respiration and replication.^9^,^10^ Additional destabilization of the membrane is caused by reactive functional groups, particularly the positively charged amino groups of the dendronized NPs.^23^ Thus, they can often create a path for antibacterial proteins to digest bacterial peptidoglycans located between the outer and inner membranes of gram-negative bacteria.^25^

Antimicrobial proteins (AMPs), which include lysines (eg, lysozyme and endolysin), have enzymatic properties that involve the lysis of peptidoglycan components. In Gram-positive bacteria, the peptidoglycan layer is located on the outside, facilitating degradation by endolysin.^26–28^ However, challenges arise for gram-negative bacteria because of their additional outer membrane, which hinders endolysin access. To address this limitation, dendritic metal NPs are often combined with endolysins to form dual-acting complex.^29^,^30^ In our previous study, we investigated the hybrid electrospun nanomat based on L-lactide-block-ε-caprolactone copolymer (PL-b-CL) mixed with polyvinylpyrrolidone (PVP) homopolymer loaded with AuNPs. Dendronized silver nanoparticles (Dend-AgNPs) were adsorbed onto the nanofibers and enhanced their antibacterial properties against *P. aeruginosa*. In addition to low cytotoxicity, it only slightly increased immunoreactivity without reducing the proliferation rate of fibroblasts.^13^ However, the antibacterial effect against gram negative bacteria is required to be even better.

Therefore, loading the dendritic metal NP/lysin complex into a nanomaterial may lead to the creation of a nanomaterial with unique properties as a dressing aimed mainly at infections with gram-negative bacteria, which belong to the group of antibiotic-resistant bacteria. Additionally, attention should be paid to its low cytotoxicity towards skin cells, which accelerates wound healing.

For this purpose, we have developed electrospun nanofibers loaded with a dendronized AgNP/endolysin complex with triple action against the multidrug-resistant gram-negative bacterium Pseudomonas aeruginosa, which inhibits biofilm formation and inhibits the growth and destruction of planktonic bacteria. Moreover, the cytotoxic properties of the developed nanomat were tested to assess its potential use as an innovative dressing material.

## Materials and Methods

### Synthesis of Electrospun Nanofibers

The nanofibers were fabricated as previously described in Lasak et al, 2024.^13^ Copolymer of L-lactide-ε-caprolactone (PLCL) was obtained from Purasorb, Corbion, Netherlands. Whereas PVP (M_w_ 250) and analytical grade chloroform used to dissolve polymers were purchased from Carl Roth GmbH, Karlsruhe, Germany. For preparing an electrospinable solution, 17% w/v PLCL polymer was dissolved in chloroform. To this solution, 0.21g PVP was added to improve the electrospinability of the solution. The solution was transferred to a 5mL syringe and placed on the programmable pump with a flow rate of 0.8mLhr^−1^. At the spinneret end a metal capillary (0.8mm inner diameter) was connected via a PTFE tube for transferring the solution from the syringe. The metal capillary was charged with +12kV leading to the formation of Taylor’s cone, and eventually, a jet could be observed moving towards the collector connected to a negative potential of −4kV. The electrospinning was performed in a closed climate-controlled chamber with an average temperature of 16°C and relative humidity of 85%. The fibers were peeled from the collector for further analysis and application.

### Dendronized Silver Nanoparticles (Dend-AgNPs)

**Figure 1:**
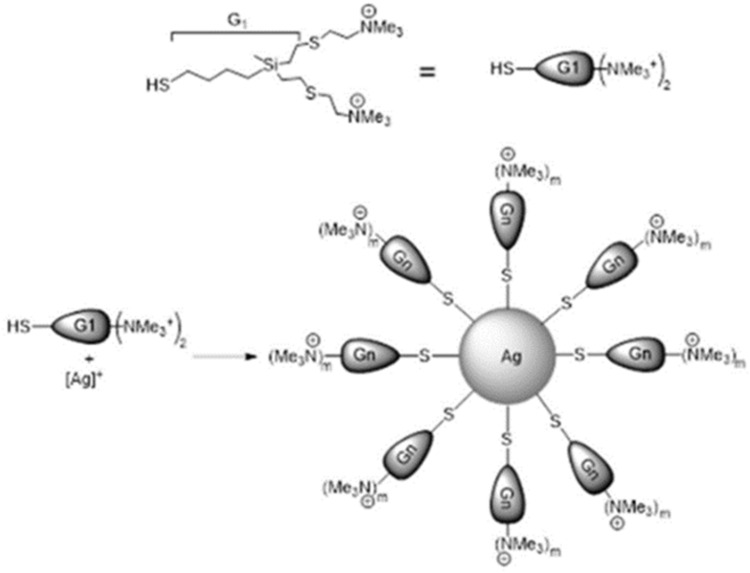
Reaction schemes of Dend-AgNP synthesis.

Dend-AgNPs were prepared as previously described elsewhere^24^ (Figure 1). Dendronized AgNPs were formed in water by the reaction of AgNO3, a small cationic carbosilane dendron, HSG1(S-NMe^3+^)_2_,^31^ and NaBH_4_, which acted as the reducing agent. The Ag/dendron/NaBH4 ratio was 1/1/5. The Dend-AgNPs were purified by dialysis (MWCO 10 K) for three days.

### Recombinant Endolysin

The recombinant CHAP endolysin fragment of the phage Î¦812 catalytic domain was purchased from Protean, Czech Republic.

### Morphology of Nanofibers

The morphologies of the unmodified and modified nanofibers were determined using scanning electron microscopy (SEM, JEOL JSM-7100F, Belgium) at an accelerating voltage of 15 kV. The samples were placed on carbon-coated 300 mesh copper grids, left to dry completely, coated with gold spray using a JEOL JFC 110E Fine Coat Ion Sputter, and analyzed by SEM. Dendronized silver nanoparticles at concentrations of 1.6 mg/mL were placed on nanofiber discs in an amount of 4 µL and left to dry for 15 minutes. Then, endolysin at a final concentration of 3 µg/mL in a volume of 1.2 µL was spotted on the dried disc with Dend-AgNPs and left to dry for 10 min. Subsequently, the discs were covered with gold using a JEOL JFC 110E Fine Coat Ion Sputter and analyzed using SEM.

### Antimicrobial Assay and Measurement of Pyocyanin and Pyoverdin

The antimicrobial activity of the nanofiber alone and the complex with Dend-AgNPs+endolysin was carried out using wild-type *P. aeruginosa* PAO1 and evaluated spectrophotometrically using an optical density test (OD 600). The levels of the dyes produced by PAO1, pyocyanin, and pyoverdin were measured. The level of pyocyanin production was measured at a wavelength of 691 nm, whereas the level of pyoverdin production was measured by fluorescence using an Ex/Em wavelength of 405/460 nm. In addition, antimicrobial activity was measured in an exponentially growing culture of PAO1 using spectrometry at a wavelength of 600 nm. The effect was expressed as absorbance of the *P. aeruginosa* culture (optical density). A TECAN SPARK Magellan V2.2 STD spectrometer (Tecan Group Ltd., Switzerland) was used. Each measurement was performed after 24 h of incubation with the test compounds, and the results represent the percentage of the control. The experiment was repeated three times.

### Morphology of Bacteria Cells Incubated with Nanofibers by SEM Microscopy

Morphology of wild-type *Pseudomonas aeruginosa* PAO1 after nanofiber (NF) (area ≈ 0.3 cm^2^) unmodified and modified, with NPs (Dend-AgNPs), or endolysin, and NPs complex (DendAgNPs+endolysin) was verified by Scanning Electron Microscopy (JEOL JSM-7100F, Belgium) at 15 kV accelerating voltage. Bacteria was centrifuge (9000 rpm, 3 min), washed twice in PBS, fixated in 3% glutaraldehyde in 0.2 M cacodylic buffer for 1 h, washed twice in 0.1 M cacodylic buffer, pre-fixated in 1% OsO_4_ in 0.1 M cacodylic buffer for 60 min, washed twice in 0.1 M cacodylic buffer, dehydrated in increasing ethanol concentration (50, 60, 70, 80, 90, 99.9%) for 10 min each, incubated in solution of ethanol and propylene oxide 1:1 for 15 min, and suspended in pure propylene oxide. The samples were placed on carbon-coated 300 – mesh copper grids, left to dry completely, covered with gold using a JEOL JFC 110E Fine Coat Ion Sputter, and analyzed by SEM.

### Bacteria LIVE/DEATH Assay

The bacterial viability under the conditions presented above was examined by fluorescence microscopy using a LIVE/DEAD BacLight Bacterial Viability Kit (Invitrogen, ThermoFisher). Dead bacteria and those with damaged membranes were stained red with propidium iodide, whereas living cells were stained green with SYTO 9. Experiments were conducted in accordance with the manufacturer’s recommendations. *P. aeruginosa* PAO1 was used in this experiment. A different combination of unmodified and modified NPs, Endolysin, was placed in a 96-well plate along with the bacterial suspension. The experiments were performed in triplicates. Viability was examined in an exponentially growing PAO1 culture after 24 h of incubation. The dyes were mixed in a 1:1 ratio. After 24 h, the nanofibers were removed from the well and the entire liquid was poured into suitable Eppendorf tubes. The liquid was centrifuged (9000 rpm/ 10 min) and the supernatant was removed and washed twice with 0.85% NaCl. Finally, the pellet was resuspended in 0.85% NaCl, the dye mixture was added and incubated for 15 min in the dark. The solution was applied to a base slide, covered with a coverslip, and then observed using a fluorescence microscope (Zeiss Axio Scope.A1) and ZEN microscopic analysis program using filters (green set 44: excitation BP 475/40, FT 500, emission BP 530/50; red set 20: excitation BP 546/12, FT 560, emission BP 575–640).

### Biofilm Inhibition Assay

Inhibition of *P. aeruginosa* biofilms was assessed using a crystal violet (CV) assay. This method quantifies the total biofilm mass based on the binding ability of the CV dye to extracellular polymeric substances (EPS) and both live and dead bacterial cells. The 24 h biofilms were gently washed twice with phosphate-buffered saline (PBS, pH 7.4) to remove unbound bacteria. PBS was removed, and the biofilms were left to dry. Next, the biofilms were stained for 15 min with 0.1% crystal violet solution and then washed three times with PBS to remove the excess dye. Next, CV was dissolved in 99.9% ethanol. After 15 min of incubation, the solution was transferred to a new 96-well plate, and the absorbance at 570 nm was measured using a TECAN SPARK Magellan V2.2 STD spectrometer (Tecan Group Ltd., Switzerland). The optical density of the de-staining solution represents the biofilm mass.

### Cytotoxic Effects on Fibroblasts

The human fibroblast cell line, VH10 (ATCC, Manassas, VA, USA; catalogue no. PCS-201-012) were maintained and cultured in medium (Fibroblast Growth medium, Cat no. 116–500, Cell Application, Inc). at 37 °C in a humidified atmosphere with 5% CO_2_. The culture medium was changed every 2 days. The viability of fibroblast cells treated with nanofibers and/or dend-AgNPs was determined using an MTS assay. The fibroblasts were seeded in 96-well plates. After reaching the appropriate confluence, the cells were treated with NF, Dend-AgNPs, endolysin, NF+Dend-AgNPs, NF+endolysin, Dend-AgNPs+endolysin, and NF+Dend-AgNPs+endolysin for 24 h. The MTS Cell Proliferation Assay Kit (Colorimetric) (Abcam) was used according to the manufacturer’s recommendations, and absorbance at 490 nm was measured using a TECAN SPARK Magellan V2.2 STD spectrometer (Tecan Group Ltd., Switzerland).

## Results and Discussion

### Preparation and Physicochemical Characterization of Nanofibers Loaded with Dend-Ag NPs and Endolysin

**Figure 2:**
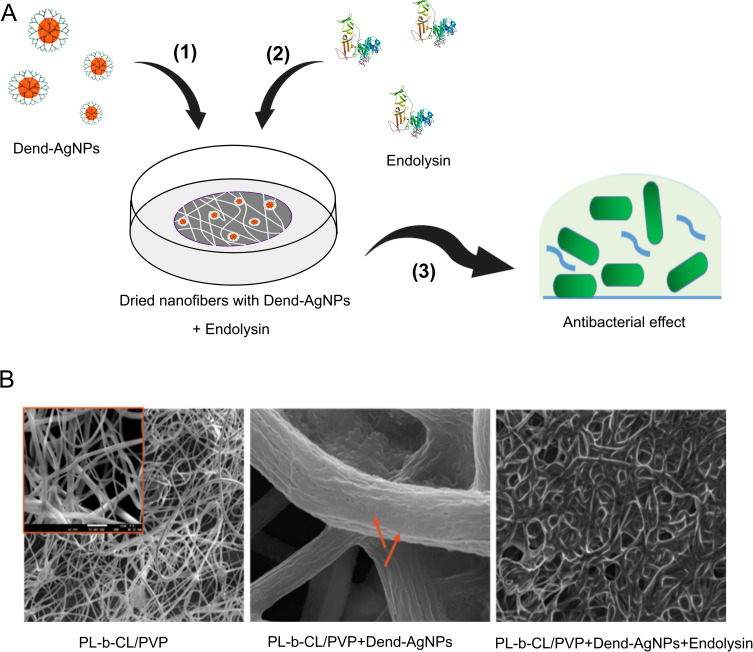
Schematic presentation of preparing nanofibers (**A**). Preparation and condition of experiment in text. (**B**) SEM photos of unmodified nanofibers (PLCL/PVP) (left), nanofibers modified with dendronized silver nanoparticles (PLCL/PVP + Dend-AgNPs) (middle) and nanofibers modified with dendronized silver nanoparticles and endolysin (PLCL/PVP + Dend-AgNPs + Endolysin) (right).

In the first stage, previously prepared UV-sterilized nanofibers were used to load dendronized silver nanoparticles (Dend-Ag NPs) and endolysin. Dend-Ag NPs were loaded into the nanofibers at a volume of approximately 4 µL at a concentration of 1.6 mg/mL. This resulted in a final concentration of approximately 32 µg/mL after the placement of nanofibers in 200 µL of the biological medium. The modified nanofibers were left to dry and the solvent containing the nanoparticles was evaporated. The nanofibers had a burgundy color. Next, 4 µL endolysin was added to the nanofibers. This step led to a concentration of 5 µg/mL in 200 µL of biological medium (Figure 2A). After a few minutes, the NanoMat was used for the experiments.

Figure 2B shows the SEM images of the PLCL/PVP nanofiber structure alone (left), with Dend-AgNPs (middle), and with Dend-AgNP-endolysin modification (right). The addition of the dendronized nanoparticles did not change the morphology of the nanofibers; however, some lumps resulting from the attached nanoparticles were visible. An interesting photograph was obtained after the addition of endolysin. The morphology of the nanofibers changed. It was easy to observe the “film” of endolysin on nanofibers. This may be due to attractive interactions between the attached nanoparticles and proteins, forming an additional scaffold between the electrospun nanofibers. However, this does not lead to visible deformation or shrinkage of the NanoMat. After placing NanoMat in a biological medium, the morphology of the nanofibers returned to its previous state because of the release of endolysin into the solvent.

### Antimicrobial Properties of Nanofibers Against Pseudomonas aeruginosa

**Figure 3:**
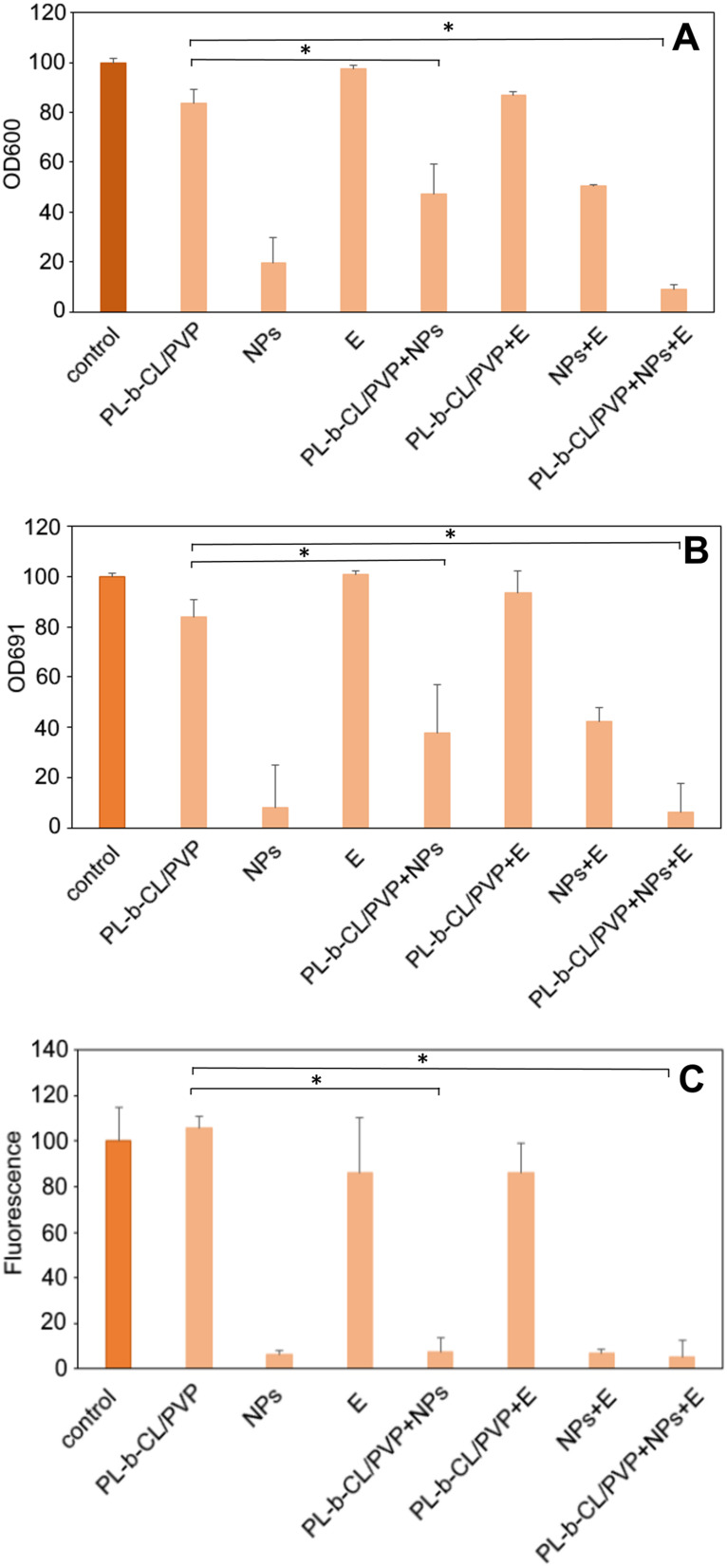
The optical density of Pseudomonas aeruginosa PAO1 measured at 600 nm without and in the presence of unmodified and modified nanofibers, Dend-AgNPs, Endolysin and Dend-AgNPs + Endolysin after 24h incubation (**A**). Pyocyanin production of Pseudomonas aeruginosa PAO1 measured at 691 nm without and in the presence of unmodified and modified nanofibers, Dend-AgNPs, Endolysin and Dend-AgNPs + Endolysin after 24h incubation (**B**). Pyoverdine production of Pseudomonas aeruginosa PAO1 without and in the presence of unmodified and modified nanofibers, Dend-AgNPs, Endolysin and Dend-AgNPs + Endolysin after 24h incubation (**C)**. Untreated cells were used as control. * p < 0.05 (PL-b-CL/PVP+NPs+E versus PL-b-CL/PVP) and (PL-b-CL/PVP+NPs versus PL-b-CL/PVP).

The antibacterial properties of the nanofibers produced against the antibiotic-resistant gram-negative pathogen, *P. aeruginosa* PAO1, were tested. Figure 3A (OD 600) shows the inhibition of bacterial growth after 24-h treatment of with nanofibers. The PLCL/PVP nanofibers reduced slightly the growth of *P. aeruginosa* ~83% of control. Additional modification of the nanofibers with Dend-Ag NPs resulted higher inhibition of bacterial growth up to 47%. NF modification with endolysin slightly reduced bacterial growth to 87%. In addition, modification with both compounds (Dend-AgNPs and endolysin) resulted in the inhibition of bacterial growth to ~9% compared to the untreated control. In contrast, similar assays without NF showed that bacterial growth in the presence of Dend-AgNPs alone was inhibited by 13%, in the presence of endolysin, no significant effect on bacterial growth, and The simultaneous presence of Dend-AgNPs and endolysin inhibited bacterial growth to 50%. Clearly, the combination of Dend-AgNPs and endolysin was the most active system when incorporated into the nanofibers.

Next, the production of two key pigments and virulence factors produced by *P. aeruginosa* - pyocyanin (Figure 3B) and pyoverdin (Figure 3C) were checked. The reduction in these factors indicated the blocking of important metabolic pathways in *P. aeruginosa*.^32–34^ The presence of unmodified nanofibers reduced pyocyanin production to a level of 84%, whereas with modification of Dend-AgNPs, production dropped to ~38%, and modification with endolysin showed no significant effect on pigment production. However, nanofiber modification with dend-AgNPs and endolysin resulted in a decrease in production to 6%. It should be noted, the presence of the dendrimer alone caused a drop in production to 8%, whereas the presence of endolysin alone had no effect on pyocyanin production.

In contrast, the presence of nanofibers did not affect the pyoverdine production (Figure 3C). In contrast, modification of nanofibers with Dend-Ag NPs caused a significant decrease in the production of the dye, up to 6%), modification with endolysin caused a slight decrease to 86%, and modification with both compounds caused a decrease in the production of pyoverdine to 5%. It should also be noted that the presence of nanoparticles alone caused a decrease in pyoverdine to 6%, in the presence of endolysin alone, it was preserved at 86%, and in the presence of both compounds, the level was at 7%. Considering the above observations, we found that the Dend-AgNP/endolysin complex is responsible for a significant reduction in the bacterial growth and production of key pigments by *P. aeruginosa*, such as pyocyanin and pyoverdin. This suggests an additional mechanism of direct destruction of the bacterial outer membrane and cell wall in addition to inhibiting certain metabolic processes in cells that lead to reduced bacterial growth, which reduces their ability to maintain infection and promotes the inflammatory process, thus confirming antibacterial properties.^10^,^13^,^35^

**Figure 4:**
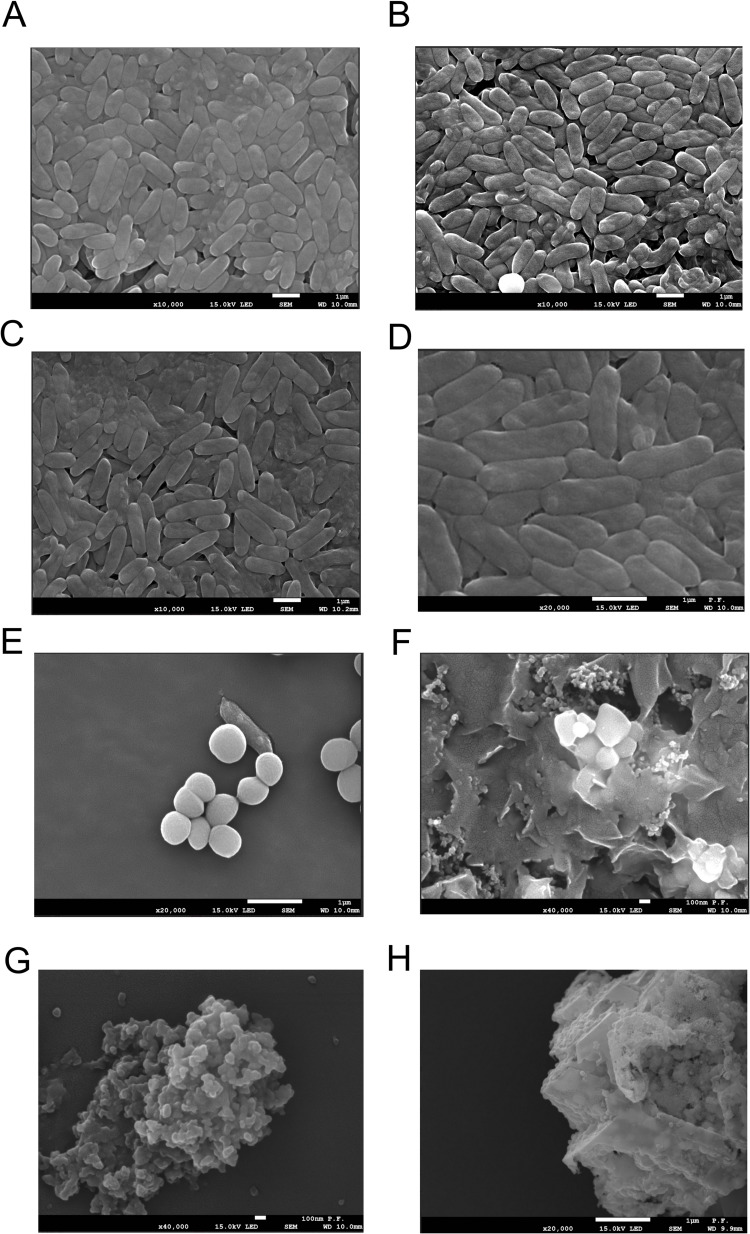
The SEM picture presents the morphology of bacteria cell untreated (**A**), treated nanofiber NF (**B**), endolysin (**C**), NF+endolysin (**D**), Dend-AgNPs (**E**), NF+Dend-AgNPs (**F**), Dend-AgNPs+endolysin (**G**) and NF+Dend-AgNPs+endolysin complex (**H**). The scale bar is 1 µm.

Scanning electron microscopy (SEM) was used to observe bacterial morphology after incubation with NanoMat (Figure 4).

The morphology of the bacterial cells remained intact and a normal structure was observed. In contrast, image E (bacteria treated with Dend-AgNPs alone) shows shrunken round bacterial cells. No bacteria-like shape was observed in photographs F (bacteria treated with nanofibers and nanoparticles), G (bacteria treated with nanofibers and endolysin), and H (bacteria treated with completed NanoMat), which may indicate the complete destruction of cells and the presence of debris Dend-AgNPs. Dendronized AgNPs alone, in complex with endolysin, and modified Dend-AgNPs+endolysin nanofibers inhibited bacterial growth by altering their cell wall morphology. The interaction of these complexes with bacterial cells causes leakage of the cell wall, release of cellular components, and production of ROS.^36^ These actions are responsible for the inhibition of bacterial growth. In general, the toxicity of NPs to bacteria is due to loss of cellular integrity, cell wall disruption or physical damage.^36–38^

**Figure 5:**
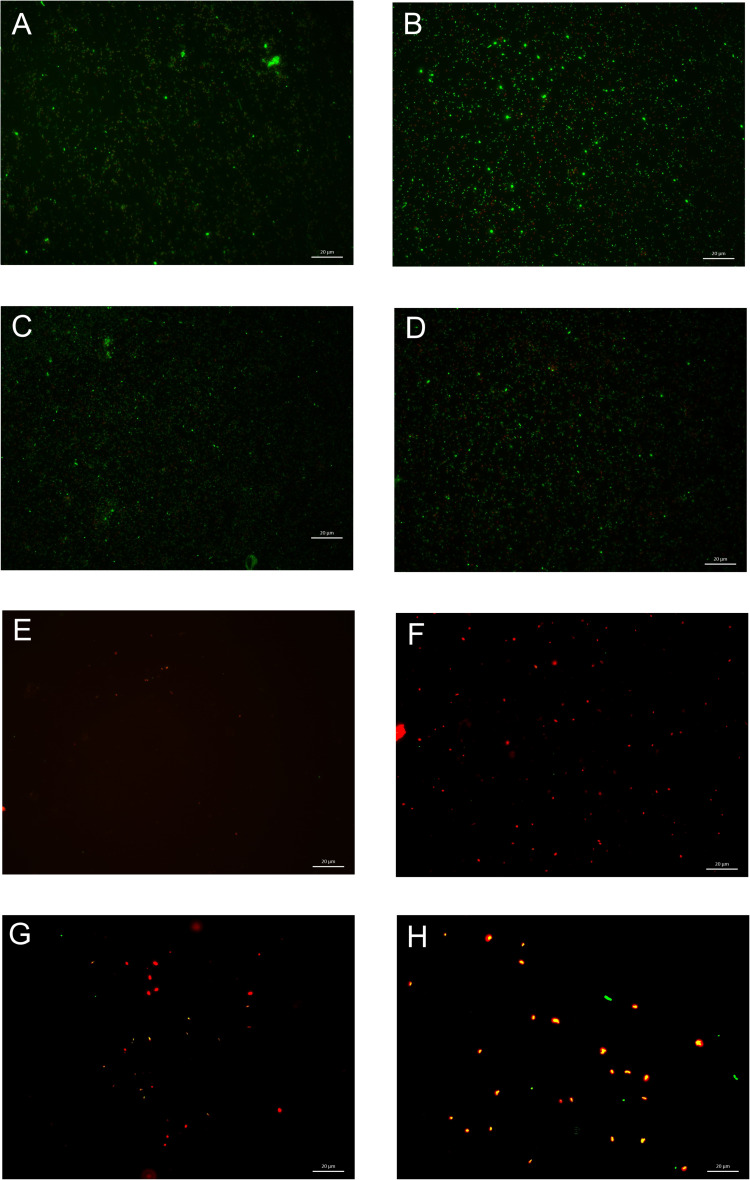
LIVE/DEAD staining results Untreated bacterial culture was used as control (**A**), after 24h treatment with unmodified nanofiber NF (**B**), with endolysin (**C**), with modified NF with endolysin (**D**), with Dend-AgNPs (**E**), with modified NF with Dend-AgNPs (**F**), with Dend-AgNPs+endolysin (**G**) and modified NF with Dend-AgNPs+endolysin complex (**H**). The scale bar is 20 µm.

To confirm the reduced viability of bacteria due to nanofibers, a LIVE/DEAD assay was performed (Figure 5). Figure 5A shows images of bacteria treated with unmodified and modified nanofibers obtained using fluorescence microscopy (LIVE/DEAD assay). Figure 5A shows the untreated bacteria used as a control. Figure 5B shows living bacteria in the presence of the unmodified nanofibers. A similar effect was observed for bacteria in the presence of endolysin (Figure 5C) and in nanofibers with endolysin (Figure 5D). Figure 5E, on the other hand, shows bacteria in the presence of Dend-AgNPs: no visible green living bacteria; only red dots suggesting dead bacteria are present. A similar image was obtained for nanofibers modified with Dend-Ag NPs (Figure 5F), Dend-AgNPs complexed with endolysin (Figure 5G), and nanofibers modified with both compounds (Dend-AgNPs+endolysin) (Figure 5H). The nanofibers modified with the Dend-Ag NP-endolysin complex showed a significant reduction in the number of living cells and a concomitant increase in the number of dead cells, indicating strong antimicrobial synergy.

All the above methods definitively indicate the great antimicrobial effect of the Dend-Ag NP/endolysin complex that is not embedded in the nanofibers. Moreover, the obtained results suggest that the pristine NFs have no significant antibacterial effect.

Previous tests were performed to evaluate antimicrobial activity against planktonic bacteria. However, it must be note that *P. aeruginosa* can create a biofilm. A bacterial biofilm is a complex structure of microorganisms embedded in the extracellular matrix that provides protection and promotes survival under adverse conditions, such as the presence of antibiotics or the immune system. Biofilms often found in wounds contribute to their chronicity, impeding healing and increasing the risk of infection.^39^,^40^

**Figure 6:**
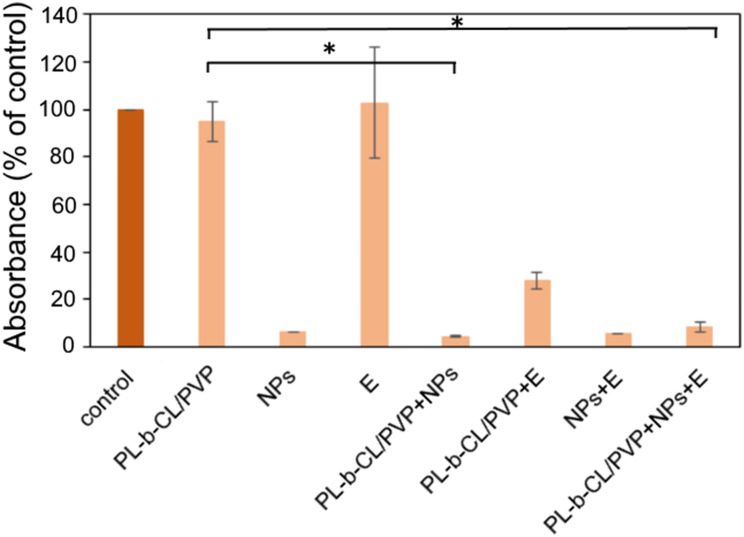
The inhibition of biofilm formation was measured as crystal violet absorbance without and with the presence of unmodified and modified nanofibers with Dend-AgNPs and/or endolysin after 24 h incubation. * p < 0.05 (PL-b-CL/PVP+NPs+E versus PL-b-CL/PVP) and (PL-b-CL/PVP+NPs versus PL-b-CL/PVP).

The inhibition of biofilm formation by P. aeruginosa PAO1 with the different nanomaterials described here is shown in Figure 6. However, nanofibers and endolysin alone did not inhibit bacterial biofilm formation. However, modification of the nanofiber with Dend-AgNPs significantly inhibited biofilm formation to 4.5%, with endolysin to 28%, while modification with both Dend-AgNPs/endolysin complexes inhibited biofilm formation to ~9%. It is worth noting that the presence of the endolysin complex and Dend-AgNPs inhibited biofilm formation to a level of 5%. In addition to directly damaging cells and limiting their growth, the suggested mechanism to explain the reduction in biofilm formation is the binding of silver ions to cysteine residues present in several enzymes associated with exopolysaccharide metabolism, which in turn may decrease or inhibit bacterial adhesion to the matrix or disrupt quorum sensing. Additionally, exopolysaccharide as the main component of the EPS matrix is mainly negatively charged, which facilitates the attachment of positively charged nanoparticles.^41^,^42^

### Cytotoxicity of NanoMat

**Figure 7:**
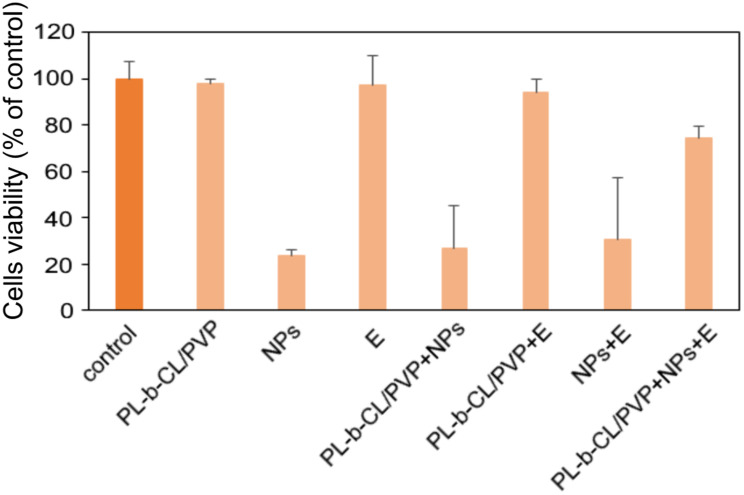
The cells viability of VH10 cells treated with unmodified and modified PLCL/PVP nanofibers, Dend-AgNPs, Endolysin and Dend-AgNPs + Endolysin after 24h incubation. Untreated cells were used as control. Experiments were performed in triplicate and results are presented as a percentage of control.

Nanomaterials used in wound healing must possess antimicrobial properties; however, additional aspects, including cytotoxicity to the skin tissue, must be considered. Therefore, the viability and proliferation capacity of fibroblast cells in the presence of unmodified nanofibers and nanofibers modified with silver dendrimers and endolysins have been studied. The results of the MTS (Figure 7) test indicated a low cytotoxic effect of the unmodified nanofibers (~98% of viability) and endolysin alone ~98%). In contrast, the presence of Dend-Ag NPs showed ~24% survival of VH10 cells. Cell viability in the presence of nanofibers modified with Dend-AgNPs reduces viability to 26%. A similar effect was observed with the dend-AgNP/endolysin complex. Unexpectedly, nanofibers with nanoparticles and endolysin showed a low cytotoxic effect of 74% compared to the control. Taken together, these results indicate that the triple nanofiber Dend-Ag NP/endolysin nanomat not only exhibits strong antimicrobial activity, but also shows reduced cytotoxicity against human fibroblasts, making it a promising safe and effective medical application in wound therapy. A complex of nanoparticles with endolysin without nanofibers also shows excellent antimicrobial properties; however, it possesses relatively high cytotoxicity. It seems that nanofibers help cells to proliferate, and embedded NPs and endolysin into nanofibers led to slower release^13^ and more effective killing of bacteria without a high cytotoxic effect.

## Conclusion

The present study highlights the significant potential of using PL-b-CL/PVP nanofibers enriched with dendritic silver nanoparticles (Dend-AgNPs) and the antibacterial protein endolysin as a modern approach to combat bacterial resistance in potent wound-healing bandages and reduce the risk of surgical site infection (Table 1). The nanofibers of PL-b-CL/PVP were modified with Dend-AgNPs/endolysin complex. In comparison with the nanomat without endolysin, the obtained antibacterial properties are much more visible. The presented material exhibited effective antimicrobial activity against wild-type Pseudomonas aeruginosa PAO1 bacteria, which are resistant to many antibiotics, effectively reducing their proliferation and production of pathogenically relevant pigments. The proposed mechanism of action is based on the damage of the outer bacterial membrane by dendritic silver nanoparticles and increasing the permeability to endolysin to digest peptidoglycan. Additionally, the use of these modified nanofibers as a support medium for dend-AgNPs significantly reduced their potential toxicity to human fibroblast cells, suggesting their potential safety in medical applications. These results underscore the prospects for using such a configured complex as a dressing material that not only counteracts infections, but also promotes tissue regeneration. A platform with such properties could be used in the future as a basis for the production of new bone cells and the creation of new blood vessels. In this way, it facilitates the manipulation of cell behavior and thus facilitates tissue regeneration. Due to the ease of preparation of nanomaterials and their properties, the most reasonable clinical application may be to reduce pathogen colonization and promote wound healing in open wounds. To fully exploit the potential of this discovery, further studies are necessary to thoroughly understand the action of this complex and confirm its efficacy and safety in various clinical applications, especially in terms of inducing an immune response, to ensure ideal biocompatibility.

**Table 1:** Comparison of Antibacterial and Cytotoxic Properties

	Bacteria Growth (Percentage of Control)	Inhibition of Biofilm Formation (Percentage of Control)	Fibroblast Cell Viability (Percentage of Control)
Nanofibers	83%	92%	98%
Dend-Ag NPs	13%	10%	24%
Endolysin	100%	100%	98%
Nanofibers + Dend-Ag NPs	47%	**4.5%**	26%
Nanofibers+ Endolysin	87%	28%	92%
Dend-ag NPS + Endolysin	50%	**5%**	39%
Nanofibers + Dend-Ag Nps + Endolysin	**9%**	**9%**	**74%**
